# Revisiting artificial intelligence diagnosis of hepatocellular carcinoma with DIKWH framework

**DOI:** 10.3389/fgene.2023.1004481

**Published:** 2023-03-07

**Authors:** Xiaomin Shen, Jinxin Wu, Junwei Su, Zhenyu Yao, Wei Huang, Li Zhang, Yiheng Jiang, Wei Yu, Zhao Li

**Affiliations:** ^1^ State Key Laboratory for Diagnosis and Treatment of Infectious Diseases, National Clinical Research Center for Infectious Diseases, Collaborative Innovation Center for Diagnosis and Treatment of Infectious Diseases, Zhejiang Provincial Key Laboratory for Drug Clinical Research and Evaluation, The First Affiliated Hospital, Zhejiang University, Hangzhou, China; ^2^ School of Computer Science, The University of Sydney, Sydney, NSW, Australia; ^3^ School of Computer Science, King’s College London, London, United Kingdom; ^4^ Department of Gastroenterology II, The First Affiliated Hospital, Zhejiang University, Hangzhou, China; ^5^ Clinical Medicine, Nanjing Medical University, Nanjing, China; ^6^ School of Computer Science, Zhejiang University, Hangzhou, China

**Keywords:** artificial intelligence, machine learning, deep learning, hepatocellular carcinoma, DIKW framework

## Abstract

Hepatocellular carcinoma (HCC) is the most common type of liver cancer with a high morbidity and fatality rate. Traditional diagnostic methods for HCC are primarily based on clinical presentation, imaging features, and histopathology. With the rapid development of artificial intelligence (AI), which is increasingly used in the diagnosis, treatment, and prognosis prediction of HCC, an automated approach to HCC status classification is promising. AI integrates labeled clinical data, trains on new data of the same type, and performs interpretation tasks. Several studies have shown that AI techniques can help clinicians and radiologists be more efficient and reduce the misdiagnosis rate. However, the coverage of AI technologies leads to difficulty in which the type of AI technology is preferred to choose for a given problem and situation. Solving this concern, it can significantly reduce the time required to determine the required healthcare approach and provide more precise and personalized solutions for different problems. In our review of research work, we summarize existing research works, compare and classify the main results of these according to the specified data, information, knowledge, wisdom (DIKW) framework.

## 1 Introduction

Estimates from year 2000 indicated that liver cancer will continue to be the most common human malignancy, with a case growth predicted at over 500,000 per year and, for high-risk countries, a large number of cases occurring before age 20 and lasting for decades (Bosch et al., 2004). Publicly available data from 2012, GLOBOCAN 2012, demonstrated that the number of cases exceeded 770,000 in 2012 (Maucort-Boulch et al., 2018). By 2018, the data in GLOBOCAN 2018 indicated that new cases had exceeded 840,000. These figures show the rapid growth of liver cancer cases worldwide and level of attention and medical care challenges that the disease requires (Freddie et al., 2018). In 2020, the American Cancer Society reports noted that, in the United states, there were 30,160 local cancer fatalities and 42,810 new instances of liver and intrahepatic bile duct tumors, implying a variety of clinical conditions that may accompany HCC, reflecting the potential pathophysiological heterogeneity and tenaciousness of the disease (Jemal et al., 2020).Therefore, in such a medical context, HCC has become a common and worthwhile research topic to investigate its early diagnosis and intervention and propose prognostic medical behaviors.

The conception of artificial intelligence (AI) was introduced in the 1950 s, and researchers have widely applied, and reinvented the intersection within the medical field through continuous exploration and innovation within the field, due to the intention of rising the expertise of clinicians and patients. AI technologies began to rapidly evolve in the 21st century, powering the training of machine learning (ML) and deep learning (DL) algorithms. Whether in prospective research; in the analysis of medical images, non-image data sources, non-routine problem formulation, and human-AI collaboration; or in the prevention and prediction of personalized patient intelligence solutions for major medical diseases, the intersection of AI and healthcare has been a promising research direction for the current and future healthcare field (Rajpurkar et al., 2022).

AI plays several roles in the medical industry and has demonstrated outstanding results at various levels. AI technology is currently being deployed in clinics to increase the operational efficiency of medical staff and minimize the misdiagnosis rate (Beam et al., 2018). Oncology therapy and management typically follow particular patterns. AI technology can be trained using clinical data interpreted by physicians and then used to identify or predict diseases based on new data of the same type (Dias and Torkamani, 2019). Furthermore, in the field of HCC, research teams have used DL with the aid of entire images to assist pathologists in diagnosis and prediction, achieving an accuracy rate over 88% by assessing the impact of diagnostic performance of pathologists with varying levels of expertise (Kiani et al., 2020). Consequently, AI is both required and advantageous for patients as a smart, dependable, and non-invasive diagnostic method. In this study, we briefly outline the use of AI approaches in the diagnosis of HCC and examine the benefits and drawbacks of various researchers’ findings by classifying and comparing their performance on several aspects.

The main purpose of this review is to collect, synthesize, and reallocate past and ongoing classification results (Perez and Grande, 2020; Feng et al., 2021) on the deployment of conventional models and tools in AI applications concerning serology, imaging, histopathology, proteomics, and the genetic diagnosis of HCC, combined with the use of the data, information, knowledge, wisdom (DIKW) framework to reorganize and reclassify the results provided by these studies to achieve horizontal performance comparisons at the same level to provide new ideas for vertical classification at different depths of the technology employed. The DIKW framework is used to understand the importance and conceptual limits of each layer by assigning certain qualities to the next layer. The first data layer is the most basic, and information adds a deeper level of content, knowledge adds the concept of how to use it, and the wisdom level determines when/what to use it (Fricke, 2009; Rowley, 2010), such as in ML, DL, neural networks, and big data for an early HCC diagnosis. The framework is also used to review the advantages and disadvantages by comparing the effects of different researchers’ models and multidimensional comparisons in the context of specific HCC medical fields. For instance, studies have demonstrated that the early diagnosis of HCC could improve the prognosis owing to early intervention (Singal et al., 2020). In addition, HCC can be diagnosed without a confirmatory biopsy owing to particular radiological features (Heimbach et al., 2018). Although ML algorithms, models, and packages are gradually optimized as technology advances, as the problems broaden, so do the demands on the models’ efficacy and accuracy. As the issues gradually diversify and the requirements for model effectiveness and algorithm accuracy gradually increase, algorithm requirements become higher. Consequently, surveillance techniques with a reliable and good sensitivity and specificity for early HCC remain scarce.

## 2 DIWK framework

**FIGURE 1:**
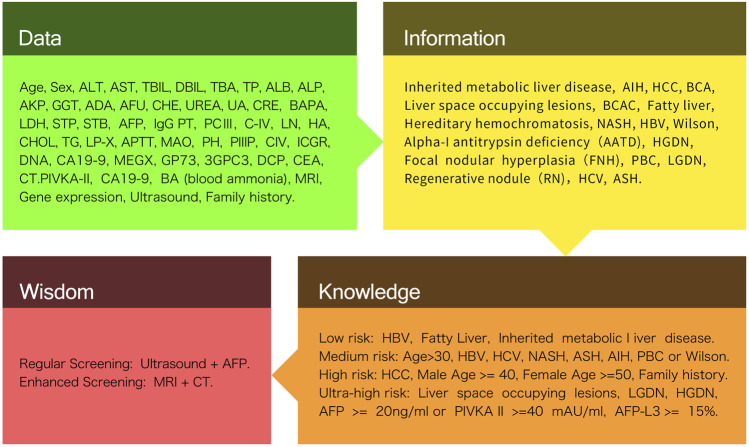
DIKW framework system overview.

This review is based on widely used research publication databases, including the Scopus, Google Scholar, and PubMed database. We started with keywords defined by articles. Because technology and medicine have developed in recent decades, these keywords were more precisely defined for certain scenarios. By searching for these keywords, we collected 83 research publications concerning similar scenarios and prepared these for a secondary filtering process. Although most of these articles covered a keyword or a combination of keywords for specific scenarios, some articles were excluded from the secondary filtering process because our focus was on the medical diagnosis and classification of HCC, such as the diagnosis of other liver diseases or the use of liver disease to determine whether other hidden diseases were present. The remaining articles were first classified by the AI and traditional medical diagnostic techniques used. Using the DIKW framework, the articles were then classified and reconstructed to obtain more relevant comparisons with similar dimensions to obtain our final review results. Here, we applied the general definition of DIKW to this review research for the specified HCC filed. In the first layer, the data contains basic testing items such as, blood testing indicators includes but not limit to, alanine transaminase (ALT), aspartate aminotransferase (AST) testing, prothrombin time (PT) testing, total-value bilirubin (TBil) blood testing, direct bilirubin (DBil) blood testing, alkaline phosphatase (ALP), albumin (ALB) blood testing, gamma-glutamyl transferase (GGT) testing, adenosine deaminase (ADA), alpha-l -fucosidase (AFU) testing, yoo-REE (urea) testing, urinary aldosterone (UA), blood ammoria (BA), lactate dehydrogenase and lactic acid dehydrogenase (LDH), superficial thrombophlebitis (STP), serum total bilirubin (STB), alpha-fetoprotein (AFP), monoamine oxidase inhibitor (MAO) testing, amino terminal peptide of type III procollagen (PIIINP), monoethylglycinexylidide (MEGX test), serum Golgi protein 73 (GP73) testing, 3-Glypican-3 (3GPC-3) testing, carbapenem-resistant Enterobacterales (CRE) testing and Immunoglobulin G (IgG) testing. Image testing data includes magnetic resonance imaging (MRI), computed tomography (CT) and Ultrasound (US). And patient’s feature indicators. The information layer refines the data from the first layer using identifiable data to perform a basic identification of the existence of HCC, using information such as Hepatitis B Virus (HBV), Hepatitis C Virus (HCV) indicators, fatty liver indicators, inherited liver disease and regenerative nodule family history. The knowledge layer fuses the valuable part of information collected through the second layer to understand the knowledge and logical connections behind the data through prerequisite knowledge. For example, by establishing the functions between the input and output, we can build ML and DL models and algorithms for prediction and derivation. In particular, if the patient’s HBC and HCV indicators are not in the normal range, the model predicts the diagnosis of HCC. Therefore, we can assume that the risk of the disease is significant given that the patient has a family history of pathology and is older. The wisdom layer, based on intelligent analysis and assisted decision making by applying a thorough understanding of the deep logic transmitted by the knowledge layer, produces reports and recommends more detailed and in-depth content such as decision-making solutions (Figure 1).

## 3 Data and information layers in the diagnosis of HCC

**FIGURE 2:**
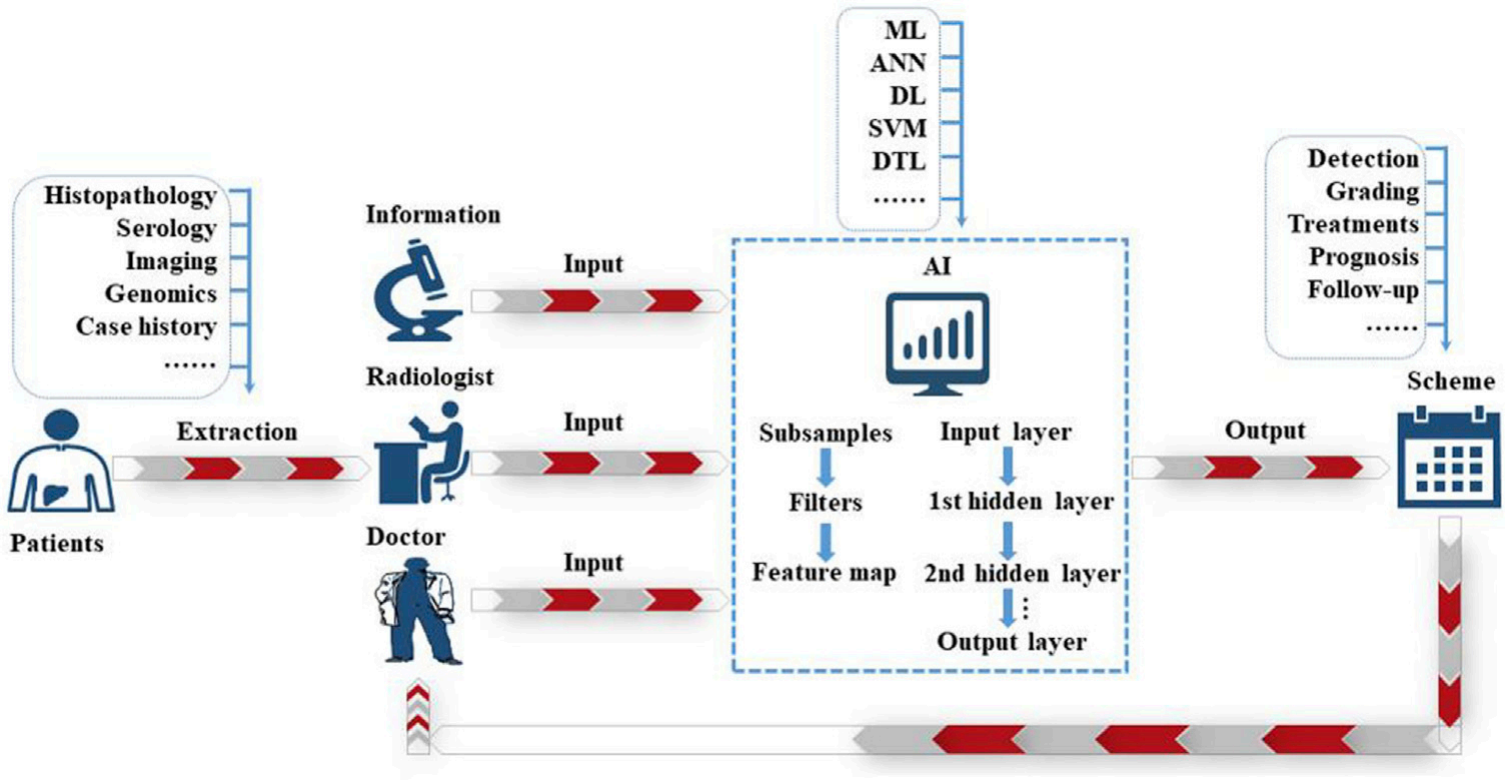
The schematic idea of AI application in the diagnosis of HCC.

Some of the common core technologies in AI include ML, NLP (natural language processing), computer vision, and robotics, which have all become autonomous sub-industries. Combined with the hot cross-domain technologies that have emerged in recent years, AI technologies have also produced different levels of quality products through their different features and advantages. Traditional ML is a type of AI based on automatically learning from previously provided data and algorithm training, to organize and recognize patterns. Support vector machines (SVMs), Bayesian networks (BNs) (Wu and Cai, 2011; Jia et al., 2015), k-nearest neighbor (KNN) (Wu et al., 2012), decision trees (DTs) (Wang et al., 2018), artificial neural networks (ANNs), and classification and regression trees have all been employed in the medicinal field (Kaul et al., 2020). Over the last decade, technological improvements have resulted in the appearance of DL as a new ML model for creating multilayer hierarchies of ANNs (Berre et al., 2020). For example, deep neural networks (DNNs) have been used in substantial research employing to handle a wide range of pattern recognition and classification tasks (Liu et al., 2020; Ma et al., 2021; Su et al., 2021), ranging from smart speakers that introduce intelligent assistants to complex computer vision tasks in self-driving automobiles (Bazrafkan and Corcoran, 2018). Many of these issues can be applied to the design of smarter consumer electronics (CE) systems and devices. Engineers must translate the results of this rich academic and industrial research into practical DNN solutions and investigate accessing the broader utility of DL such that increasingly large datasets can be processed in a reasonable time-frame in the CE industry, particularly with the arrival of optimized hardware based on graphics processing units (GPUs) (Lemley et al., 2017). AI is gaining popularity in clinical decision-making concerning HCC. Figure 2 presents an overview of applying AI to HCC diagnosis.

### 3.1 Image data and information

#### 3.1.1 Serology

The early detection of HCC is critical for improving prognosis and long-term survival. The traditional paradigm relies on imaging tests, such as ultrasonographic methods that are not as sensitive as serologic markers. Serologic markers are convenient, fast, inexpensive, and can be used for the dynamic surveillance of HCC (Witjes et al., 2013). However Serology is prone to false positives and negatives. The diagnostic potential of four serum biomarkers was used to model the diagnosis of HCC *via* multilayer perceptron (MLP) and radial basis function (RBF) neural networks, according to a combination of results from previous studies (Memarian and Balasundram, 2012; Li B. et al., 2017). The results showed that the combination of serological markers and ANNs could improve the sensitivity and accuracy of HCC diagnosis, thereby improving the prevention and treatment of HCC. The applications of AI technology can analyze the disease risk coefficient from large datasets related to the laboratory indicators of patients.

In 2005 (Wang et al., 2005), a team developed a method for detecting serum protein fingerprints using protein microarray technology, in which surface-enhanced ionization time-of-flight mass spectrometry (SELDI-TOF-MS) on protein biochips was combined with ANN analysis to analyze and model liver cancer, cirrhosis, and healthy individuals. The trained ANNs’ sensitivity and specificity for detecting liver cancer reached 88.2% and 94.6%, respectively. This optimized the sensitivity and specificity compared with the conventional approach. Unlike the aforementioned single-factor analysis, a multi-factor analysis approach can establish a higher diagnostic value (Ning et al., 2021). Xie et al. (2018) constructed an expression detection system based on the GeXP system for nine genes: GPC3, HGF, ANXA1, FOS, SPAG9, HSPA1B, CXCR4, PFN1, and CALR. The team developed a multi-parametric gene expression analysis method by combining logistic regression analysis, discriminant analysis, classification trees, and DNNs to model the diagnosis of groups of early-stage HCC patients and healthy controls by routinizing the area under the curve (AUC), sensitivity, and specificity. The specificity was used as the target function and as the final diagnostic index. The results showed that the ANN detection system was most valuable for the diagnosis of HCC with a high AUC (0.94), sensitivity (98%), and specificity (85%).

Combined with these research results, ANNs, as a major branch of machine learning, have excellent experimental feedback in both single- and multi-factor analysis, which provides confidence to researchers concerning the future cross-section of liver cancer treatment and diagnosis in the context of AI-medicine.

#### 3.1.2 Imaging

Ultrasound (US), magnetic resonance imaging (MRI), computed tomography (CT), positron emission tomography (PET), and other imaging techniques play an important role in the diagnosis and therapeutic effect assessment of HCC. Radiomics combined with AI has gradually verified to be a promising breakthrough in clinical analysis, for the precise quantitative information it provides and therefore the extra discriminatory options still unknown, gives aid professionals with a lot of correct predictions for identification pathological lesions within the liver (Feng et al., 2021). However, the abilities of CT and MRI with extracellular agents to differentiate cirrhotic nodules, dysplastic nodules, and early HCC is limited (Choi et al., 2014). Fortunately, the two-dimensional attribute and digitization trend of medical image are approved to appropriately fit an AI application. Because cancer is heterogeneous in both space and events, this limits the use of invasive biopsies based on molecular detection, but offers great potential for medical imaging. Over the past decade, innovations in medical imaging and advances in radiomics research have led to significant breakthroughs in the development of quantitative imaging. In 2012, radiomics was proposed and well adapted for use in oncology research (Lambin et al., 2012). A high-throughput extraction of a large number of image features from radiological images has solved the problems of extracting more information from image-based features, reproducible analysis methods, etc. However, further validation is required in a multicenter setting and in real clinical trials (Mokrane et al., 2020).

The aim of radiomics is to provide precise risk stratification by incorporating imaging traits into predictive models for treatment outcomes. This high-throughput approach extracts a large amount of imaging data using computer-aided engineering and creates a variety of image-based, quantifiable features to establish a connection between different characteristics and diagnosis, therapy, and prognosis (Lambin et al., 2017).

#### 3.1.3 CT

Liver segmentation in CT imaging is of great importance to assess liver lesions and plan the ideal treatment. Several studies have shown that the application of AI combined with traditional CT examination improves the diagnostic accuracy of HCC. Computed tomography texture analysis (CTTA) is a method of quantifying lesion heterogeneity to distinguish different lesions. A study compared CTTA software involving an RF model against two radiologists in the accuracy of analyzing 17 cases of focal nodular hyperplasia, 19 hepatic adenomas, 25 HCC, and 19 cases of normal liver parenchyma, demonstrating the model had a significantly higher prediction accuracy ( > 90% vs. 72.2% and 65.6%) (Raman et al., 2015).

In addition, Ouhmich et al. (Ouhmich et al., 2019) used a DL cascaded convolutional neural network (CNN) based on U-Net architecture to differentiate normal liver tissue from HCC on multiphase CT images automatically, and their proposed method was comparable to state-of-the-art methods for automatic MR image segmentation and significantly outperformed traditional interactive CT image segmentation techniques, thereby allowing for the effective assessment of the necrosis rate of tumor tissue.

Furthermore, Yamada et al. (Yamada et al., 2019) determined that the diagnostic performance of transfer learning (TL) using a pretrained CNN was robust to the error registration of multiphase HCC images (Cao et al., 2020), and they retrospectively evaluated over 200 consecutive patients with actual primary liver cancer. Their results indicated that the CNN combined with a DCE-CT graphics processing model has good effects for liver cancer prevention diagnosis by observing the diagnostic work of another research team (Yasaka et al., 2018) using DL methods and CNN to differentiate liver masses in dynamic CT scans by building a CNN model with six convolutional, three maximum pooling, and three fully connected layers to achieve a median AUC of 0.84. According to these studies, we know that the DL model deployment in CT diagnosis provides good performance results for both preventive diagnosis and error tolerance. Balagourouchetty et al. (Lakshmipriya et al., 2018) extracted the deep features of CT images with a TL method and built an integrated FCNET classifier, which could accurately classify six types of liver CT images: normal, HCC, hemangioma, cyst, abscess, and liver metastases. They reported that, based on ML techniques, using quantitative imaging features extracted from triphasic CT scans can enhance the diagnostic accuracy of HCC in cirrhotic patients with indeterminate liver nodules.

#### 3.1.4 MRI

Compared with other imaging methods, MRI is more complex owing to each irreplaceable sequence in the tissue comparison mechanism (Zhao et al., 2022). The salient appearance features in MRI and changes between different phasic images are vital clues for HCC detection, segmentation, and grading. Because of its higher resolution and contrast-enhanced function compared with ultrasound and CT, MRI is currently recommended as the preferred imaging method for diagnosing liver cancer. However, bypassing the challenge of imbalance is difficult with HCC training samples for models built based on the methods of excellent imaging tools such as MRI, because the classification performance of classification models, such as CNN, based on imbalanced samples tend to fit more sample classes, which is not conducive for obtaining generalized and effective models. The proposed relay backpropagation method can effectively retain relevant information and suppress the negative effects of less relevant information. Owing to the gradient flow of information in backpropagation and by introducing one or more intermediate output modules in the intermediate segment, a significant improvement in accuracy can be achieved (Shen et al., 2016). Based on the breakthrough of this work, Yang et al. (Yang et al., 2019b) proposed an MCF-3DCNN model that consists of five 3DCNNs with the same structure, and the collected HCC samples were reorganized into different three classes for model training to achieve a strong differentiation and diagnosis performance for wilson disease (WD) HCC, with an average AUC, accuracy, and sensitivity reaching 0.96, 91%, and 97%, respectively.

In addition, unlike classifying HCC pathologies and categories. Grading the degree of HCC is an important tool for the diagnosis and prevention of HCC using DL models, and a deeply supervised loss function to further improve the performance of lesion features was designed by the research team in (Zhou et al., 2019) by augmenting the training set with a resampling method. In particular, their study performed a 3.0 T MR scan using the diffusion-weighted image (DWI) conventional medical treatment combined with the breath-holding MRI method and a b-value log-transformation with three different levels set to obtain logarithmic maps, log_*b*_0, log_*b*_100, and log_*b*_600. Finally, a quadruple cross-validation with multiple validations of the training and test sets was performed to obtain the HCC grading results. The deep supervised loss function of this experiment yielded the highest HCC grading accuracy at the time, that is, 80%, and a relatively excellent AUC value of 0.83 for deep feature fusion. Similar to the application of DWIs on MRIs, a three-dimensional CNN was proposed (Trivizakis et al., 2018) to solve a high b-value, that is, log_*b*_1000 images for the diffusion comparison to reflect its higher diagnostic value in clinical medicine. Using different manners of classification validation, we determined that using softmax instead of an SVM can produce slightly higher accuracy results of 3%, and the deep learning architecture of a 3D network can improve the accuracy from 69% to 83%. This study also partly shows that 3D CNNs for HCC diagnosis is promising but requires further large-scale dataset validation. However, unlike the 3D MRI imaging work adopted in this study (Le et al., 2016), the ANN developed in this study was used to differentiate tumor voxels from non-tumor voxels by initially presenting a raw 3D image through MRI imaging and then using an anisotropic diffusion algorithm to extract the 3D-region of interest (ROI) from the raw 3D image. Finally, the edge potential images are used to mark those that respond to the threshold filter for the regional training of Single Hidden-layer Feedforward Neural Networks (SLFN), and the performance of the model was evaluated. It illustrated a significant improvement in accuracy and AUC comparing with the combined 2D structure of the DL model and conventional MRI diagnosis.

#### 3.1.5 Ultrasound (US) and PET-CT

US has been widely used as one of the most appropriate tools for evaluating liver disease and detecting new lesions. However, the lack of visual quality from various sources may result in increased errors in US diagnosis. For example, speckle noise and visual blurring complicate the automatic diagnosis of hepatic steatosis with US images, and interobserver variation in image interpretation may occur, and nodules with subtle lengths ( < 1 cm) may prevent US from automatically diagnosing the image (Farinati et al., 2009; Rhyou and Yoo, 2021). Although a weighted variance-based approach to decompose the self-contained arithmetic mean and determine the wavelet threshold was proposed in 2009 and 2011, which can be used to reduce speckle noise in US images or a non-local (NL) mean filter to reduce scatter in US images and preserve structural details and image edges, the challenge remains severe owing to the gradual increase in the requirement for diagnostic accuracy and diversity of pathological changes (Coupe et al., 2009; Rahman et al., 2011). The research team in (Rhyou and Yoo, 2021) developed a predictive model for fully automated liver pathological degeneration using three DL neural networks. The model applies migration learning to semantically segment the liver and kidney, and, as the neural network involves moral semantic segmentation, the liver and kidney (L-K) region is cropped from the original US image by the environment typically located around the liver and scores the severity of liver disease. The results of this experiment were comparable to those of medical experts, which showed a superior sensitivity of 99.8%, diagnostic accuracy of 99.91%, and specificity of 100%. Therefore, AI technology can increase the accuracy, sensitivity, and specificity of US for diagnosing HCC.

Fukuda et al. (Fukuda et al., 2010) established an image analyzing system based on neural networks to numerically calculate a coarse score (CS) that can serve as a useful predictor for developments against HCC. Time-intensity curve (TIC) analysis in the neural network analysis of contrast-enhanced ultrasonography (CEUS) provided fast and reliable diagnostic aid for classification of HCC (Streba et al., 2012). In recent studies, SVM and deep CNN (DCNN) technologies have been applied in the background of USs to classify benign and malignant liver focal lesions (FLL), which can significantly improve the diagnostic accuracy of imaging doctors. Kondo et al. (Kondo et al., 2017) constructed an automatic classification method based on ML in the CEUS of focal liver lesions. The results of 98 subjects indicated that the accuracy of the classification of benign, HCC, and metastatic liver tumors was 84.4%, 87.7%, and 85.7%, respectively, which were consistent with the CEUS guidelines for the diagnosis of FLL. Similarly, using ANNs to analyze liver uptake of fluorine 18 fluorodeoxyglucose (FDG) with patients’ laboratory data achieved a high sensitivity and specificity for detecting HCC (Preis et al., 2011).

### 3.2 Biological data and information

This section reviews the contribution of AI tools and applications to the prevention and diagnosis of HCC in the field of bioinformatics, which primarily focuses on three sections: histopathology, proteomics, and genomics.

#### 3.2.1 Histopathology

Histopathology diagnosis is the gold standard in diagnosing HCC with clearer features than other examinations. Formulating a treatment strategy is important; however, high standards in clinical experience and the professional skills of pathologists are required (Rajpurkar et al., 2018) (Esteva et al., 2017). AI has many advantages over human beings; AI can compute entire sections, specific tissue, and different cell types *via* intelligent algorithm. This makes establishing predictive biomarkers based on an accurate quantitative histological model possible, providing a new detection tool for oncologists and pathologists to determine the prognosis and treatment effect of patients (Coudray et al., 2018). Moreover, AI can detect imperceptible details missed by humans because of its strong objective analysis ability, particularly in the molecular characteristics of pathological sections. A research on a targeted feature model for differentiating HCC from adjacent normal tissue and predicting the prognostic response of HCC patients after surgery was presented in 2020 (Liao et al., 2020b). The targeted feature model is based on histopathological images and its main approach is to train the features extracted from tissue sections, obtain a statistical model for classification, improve it, and optimize it with the original researcher’s ML approach (Liao et al., 2020a) to predict the patient’s diagnosis and postoperative response. An AUC of 0.886 was verified in the test and external validation sets. Unlike some cancers such as lung cancer subtypes, distinguishing between, for example, adenocarcinoma (LUAD) and squamous cell carcinoma (LUSC) requires a considerably experienced pathologist to visually inspect the data. In 2018, Coudray et al. (Coudray et al., 2018) trained a DCNN using histopathological images of lung cancer obtained as full-slide images for image analysis (inception v3). Their model slightly outperformed the pathologist’s diagnosis with an AUC of 0.97. Furthermore, recent research has claimed that a CNN combined with an extreme learning machine, the CNN-ELM model, can score HCC effectively (Li S. et al., 2017). Other studies have proposed applying fractal dimensions (FDs) to HCC diagnosis based on ANNs that have demonstrated decision systems capable of differentiating the histological images of normal parenchyma from malignant parenchyma and classifying HCC and liver metastases (Gheonea et al., 2014).

#### 3.2.2 Proteomics

Proteomic analysis for the prediction and diagnosis of HCC has been studied for over 50 years: from the discovery of alpha-fetoprotein (AFP) as the first serum biomarker for HCC in 1963 (De Mees et al., 2006) to the help of ML algorithms as tools, including RF algorithms, SVMs, logistic regression, and MLP algorithms to cluster proteomes for HCC detection. This illustrates the value of studying the proteomes of these biomarkers and integrating multi-omics techniques with proteomic distribution patterns to diagnose HCC with a higher accuracy, sensitivity, and specificity (Kimhofer et al., 2015) (Moldogazieva et al., 2021) (Feng et al., 2022).

#### 3.2.3 Genomics

AI has been used to address problems in clinical genomic analysis, including variant classification and the correspondence between genotype and phenotype (Dias and Torkamani, 2019). AI applications in the genomics of HCC have been able to correctly identify the most suitable gene by training the gene expression profile to predict prognosis and recurrence. Chaudhary et al. (Chaudhary et al., 2017) built a DL-based, survival-sensitive model using RNA sequencing, miRNA sequencing, and methylation data from The Cancer Genome Atlas (TCGA). Their model could accurately and effectively predict lesions in HCC patients as well as postoperative recurrence problems. Their study used 16,000 genes obtained from RNA sequencing, 365 miRNAs from miRNA sequencing, and 20,000 genes from DNA methylation data as input features, which were then stacked by the DL framework through the histological features for self-encoder neural network training. Marsh et al. (Marsh et al., 2010) evaluated a group of allelic deletion tumor suppressor genes (1p, 3p, 5q, 7q, 8q, 9p, 10q, 17p, 17q, 18q) using an ANN model for HCC diagnosis. The combined models predicted HCC recurrence outcomes with complete accuracy. All of these technologies found above i shown on Table 1, and we distinguished these technologies into data and information layer shown in the Table 2.

**TABLE 1:** Studies in the field of diagnosis of HCC.

Research ref	Aims of the study	Diagnostic techniques	AI aspects	Algorithms/Models adopted	Sample Size(rounded)	Optimal performances
Kiani and Amirhossein et al	Liver cancer classification	Histopathology	Deep Learning	CNN	70	Accuracy: 0.885
Li et al	Early diagnosis of HCC	Serology	Deep Learning	ANN	347	AUC: 0.764 Accuracy: 0.81/Specificity: 0.855
Wang et al	Early diagnosis of HCC	Proteomics	Deep Learning	ANN	106	Sensitivity: 88.2%/Specificity: 94.6%
Xie et al	Early detection of hcc	Genomics	Deep Learning	ANN	52	AUC:0.943/Sensitivity:98%/Specificity: 85%
Raman et al	Predicting classification of HCC	Imaging CT	Machine Learning	Random Forest	80	Accuracy: 98.6%
Ouhmich, Agnus, Noblet, Heitz and Pessaux	Prediction by liver tissue segmentation	Multiphase CT	Deep Learning	CNN	7	Accuracy: 84%
Yamada et al	Classification and differentiation of primary liver cancers	Dynamic contrast-enhanced computed tomography (DCE-CT)	Deep Learning	CNN	215	Mean DPs for CNNs: 44.1%
Yasaka, Akai, Abe and Kiryu	Differentiation of Liver Masses	CT	Deep Learning	CNN	460	AUC: 0.92/Accuracy: 0.84
Lakshmipriya, Pragatheeswaran, Biju and Ramkumar	Diagnosis of different classes of liver tumour	CT	Machine Learning	SVM	634	Accuracy: 93%/AUC: 0.9959
Yang et al	Pathologic Grading to HCC	MRI	Deep Learning	MCF-3DCNN	150,000	AUC: 0.96, Accuracy: 91%, Sensitivity: 97%
Zhou, Wang, Xie and Zhang	Grading to HCC	MRI	Deep Learning	CNN	100	AUC: 0.83/Accuracy: 80%
Trivizakis et al	Classification and diagnosis of HCC	MRI	Deep Learning	3D-CNN	130	Accuracy: 83%
Le et al	Classification and diagnosis of HCC	MRI	Deep Learning	ANN	16	None
Rhyou and Yoo	Prediction of HCC diagnosis	Ultrasound	Deep Learning	ANN	3,200	Sensitivity: 99.8%/Accuracy: 99.91%/Specificity: 100%
Fukuda, Ebara, Kobayashi, Sugiura and Yahagi	Prediction of HCC diagnosis	Ultrasound	Deep Learning	ANN	56	None
Kondo et al	Classification and diagnosis of HCC	Ultrasound	Machine Learning	Rondom Forest	98	Sensitivity: 94.0%/Accuracy: 87.7%/Specificity: 87.1%
Preis, Blake and Scott	Classification and diagnosis of HCC	PET/CT	Deep Learning	ANN	98	AUC: 0.905
Liao et al	Classification and diagnosis of HCC	Histopathology	Machine Learning	Rondom Forest	1733	AUC: 0.886
Li, Jiang and Pang	Classifying and Grading to HCC	Histopathology	Deep Learning	DCNN	83	AUC: 0.97
Ward et al	diagnosis of HCC	Proteomics	Deep Learning	ANN	144	Sensitivity: 94%/Specificity: 86%/AUC: 0.92
John, Luk and Lam	diagnosis of HCC	Proteomics	Deep Learning	ANN	66	Sensitivity: 96.97%/Specificity: 87.88%
Chaudhary, Poirion, Lu and Garmire	Prediction of HCC diagnosis	Multi-omics(Genomics/Proteomics/Histopathology)	Deep Learning	ANN	360	None
Marsh et al	diagnosis of HCC	Genomics	Deep Learning	ANN	103	Accuracy: 88.3%

**TABLE 2:** Studies of Data and Information in DIKW framework.

Research ref	Aims of the study	Diagnostic techniques	AI tools	DIKW layer
Li et al	Early diagnosis of HCC	Serology	DL-ANN	Information Layer
De Mees, Bakker, Szpirer and Szpirer	Detection of HCC	Histopathology	Alpha-fetoprotein (AFP) biomarker	Data Layer
Raman et al	Predicting classification of HCC	Imaging CT	DL-ANN	Information Layer
Ouhmich, Agnus, Noblet, Heitz and Pessaux	Prediction by liver tissue segmentation	Multiphase CT	DL-CNN	Information Layer
Kiani and Amirhossein et al	Liver cancer classification	Histopathology	DL-CNN	Information Layer
Yasaka, Akai, Abe and Kiryu	Differentiation of Liver Masses	CT	DL-CNN	Information Layer
Lakshmipriya, Pragatheeswaran, Biju and Ramkumar	Diagnosis of different classes of liver tumour	CT	ML-SVM	Information Layer
Zhou, Wang, Xie and Zhang	Grading to HCC	MRI	DL-CNN	Information Layer
Liao et al	Classification and diagnosis of HCC	Histopathology	ML-RF	Information Layer
Le et al	Classification and diagnosis of HCC	MRI	DL-ANN	Information Layer
Rhyou and Yoo	Prediction of HCC diagnosis	Ultrasound	DL-ANN	Information Layer
Fukuda, Ebara, Kobayashi, Sugiura and Yahagi	Prediction of HCC diagnosis	Ultrasound	DL-ANN	Information Layer
Kondo et al	Classification and diagnosis of HCC	Ultrasound	ML-RF	Information Layer
Li, Jiang and Pang	Classifying and Grading to HCC	Histopathology	DL-DCNN	Information Layer
John, Luk and Lam	diagnosis of HCC	Proteomics	DL-ANN	Information Layer
Marsh et al	diagnosis of HCC	Genomics	DL-ANN	Information Layer

## 4 Knowledge and wisdom layers in the diagnosis of HCC

### 4.1 Knowledge in four stages

According to the structure of the knowledge and wisdom layers that we redefined for the DIKW framework, there is a close correlation in diagnosing HCC. We divide the layers into strictly four types of stages based on knowledge extracted from the DIKW framework in the field of precision medicine on nutritional epidemiologic, nursing, etc. (Gee et al., 2012; Chen et al., 2017; Yang et al., 2019a). The four stages are Low, Medium, High, and Ultra-High Risk. At Low Risk, using the knowledge of simple HBV, fatty liver, and inherited liver disease, we provide a protocol of regular screening every 12 months. When an irregular increase of AFP occurs, we advise regular screening every 6 months. We advise this regardless of whether the current risk is in the low or medium level. At High Risk, with males above 40 years old and females above 50, enhanced screening every 6–12 months and regular screening every 3–6 months is advised. The most serious is when the patient is detected with nodules, it comes to the Ultra-high risk tier in the knowledge layer, which contains key knowledge involving lesions in the liver space, low-grade dysplastic nodules (LGDN), high-grade dysplastic nodules (HGDN), and an AFP index not less than 20 ng/ml. We then advise regular screening and enhanced screening every 3 and 6 months, respectively.

### 4.2 Wisdom in screening

According to the high and ultra-high risk stages introduced in 4.1, two different screening tools are mentioned, which is a means to reflect effective, intelligent decision-making. Regular screening is performed *via* US and AFP screening for patient cycles ranging from 3 to 12 months, whereas enhanced screening is performed *via* MRI + CT for periodic examinations ranging from 6 to 12 months to provide optimized decisions and conclusion reports.

**FIGURE 3:**
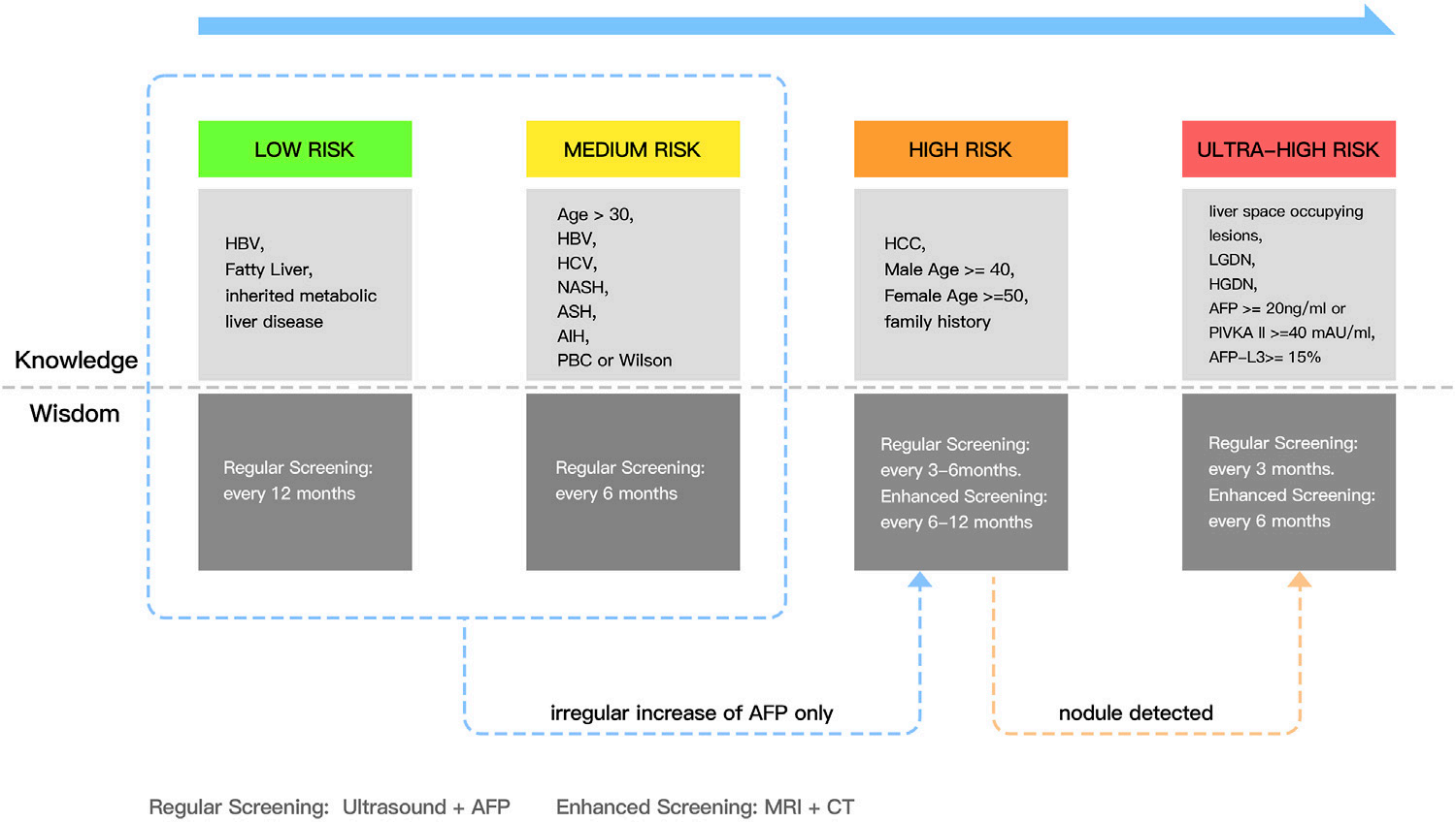
The overview of knowledge and wisdom in the diagnosis of HCC.

Evidently, the majority of the experimental methodology used in the literature and the research results produced by AI tools are mostly at the level of information and knowledge. The research output corresponding to the data layer does neither satisfies research expectations after introducing AI tools nor conforms to the requirements of synthesizing the research output to respond to more precise and accurate personalized solutions. Therefore, the large amount of literature focused on the information and knowledge layers (Figure 3) has led us to hope that AI should be applicable to tasks beyond simple feature prediction, such as in providing more comprehensive reporting output for accurate and personalized treatment plans.

First, reviewing the literature covered in this review, we found that the majority of studies were retrospective in nature, but the same protocols and models were often not adopted in diverse situations, owing to the high heterogeneity in HCC prevention and treatment. Moreover, the performance of these prediction models must be validated on large-scale and multicentered datasets. Second, we determined that most of the studies reviewed are not highly reproducible or generalizable and that solving imaging noise, multi-omics relationships, and heterogeneity is urgent in the actual medical scenario. However, the underlying mechanisms are not yet clear. Based on this discussion, we plan to create highly reusable ML and DL algorithms and models with the support of existing large-scale hospital data and then gradually extend to theoretical arguments and demonstrate the reliability of these models and objective function optimization to overcome some current limitations. These technologies of HCC diagnosis filed are matched to the layers of knowledge and wisdom respectively, see Table 3.

**TABLE 3:** Studies of Knowledge and Wisdom Layer in DIKW framework.

Research ref	Aims of the study	Diagnostic techniques	AI tools	DIKW layer
Yamada et al	Classification and differentiation of primary liver cancers	Dynamic contrast-enhanced computed tomography (DCE-CT)	DL-CNN	Knowledge Layer
Yang et al	Pathologic Grading to HCC	MRI	DL-MCF-3DCNN	Knowledge Layer
Trivizakis et al	Classification and diagnosis of HCC	MRI	DL-3D-CNN	Knowledge Layer
Xie et al	Early detection of hcc	Genomics	DL-ANN	Knowledge Layer
Preis, Blake and Scott	Classification and diagnosis of HCC	PET/CT	DL-ANN	Knowledge Layer
Ward et al	diagnosis of HCC	Proteomics	DL-ANN	Knowledge Layer
Wang et al	Early diagnosis of HCC	Proteomics	DL-ANN	Wisdom Layer
Chaudhary, Poirion, Lu and Garmire	Prediction of HCC diagnosis	Multi-omics (Genomics/Proteomics/Histopathology)	DL-ANN	Wisdom Layer

## 5 Conclusion

Based on the DIKW framework, this paper reviews the latest progress of AI technology in data, information, knowledge and wisdom of HCC diagnosis. First, among them, more than 33 related works only stay at the layer of data and information, of which 16 are related to HCC detection, it makes the inspiration and potential value of data not be fully explored. Second, the technology at the level of knowledge and wisdom is relatively rare, including only 12 cases, while only 8 cases are related to HCC assistance and detection. It is worth noting that only two cases of HCC AI auxiliary medical treatment at the level of Wisdom are included, This shows that there is still a lot of space for further exploration in the direction of HCC AI assisted medical treatment. For example, multi-modal AI algorithm can appropriately apply the fusion of multi-dimensional information. Information fusion is used to integrate image, text, gene and other information to better help the knowledge layer establish a complete knowledge graph. At present, we have not yet observed the establishment of any knowledge graph about HCC AI medical assistance information, and the knowledge graph based on multi-modal information fusion can provide more comprehensive and accurate diagnosis and decision-making basis for medical experts in assisting decision-making at Wisdom level.

However, most of the current artificial intelligence models are developed using retrospective training data collected from a single center, makes inappropriate experiment. Therefore, in the era of big data, multi-group data and next generation sequencing technology are expected to further improvement of accuracy of HCC AI diagnosis.
